# Whole-Genome Sequencing of Nontyphoidal Salmonella enterica Isolates Obtained from Various Meat Types in Ghana

**DOI:** 10.1128/MRA.00033-19

**Published:** 2019-04-11

**Authors:** Moon Y. F. Tay, Frederick Adzitey, Stella Amelia Sultan, Joseph Makija Tati, Kelyn L. G. Seow, Joergen Schlundt

**Affiliations:** a School of Chemical and Biomedical Engineering, Nanyang Technological University (NTU), Singapore; b Nanyang Technological University Food Technology Centre (NAFTEC), Nanyang Technological University (NTU), Singapore; c Department of Animal Science, Faculty of Agriculture, University for Development Studies, Tamale, Ghana; d Department of Veterinary Science, Faculty of Agriculture, University for Development Studies, Tamale, Ghana; Broad Institute of MIT and Harvard University

## Abstract

Here, we report the draft genome sequences of 16 nontyphoidal Salmonella enterica isolates obtained from locally produced meats in Tamale, Ghana, which are commonly consumed by most natives as an important protein source. The draft genomes will help provide a molecular snapshot of Salmonella enterica isolates found in these retail meats in Tamale.

## ANNOUNCEMENT

Nontyphoidal Salmonella (NTS) strains can cause mild to moderate, mostly self-limiting gastroenteritis in humans and can be acquired through many sources, including the consumption of contaminated meat (1). It should be noted that the mortality rate typically reported for NTS strains is 0.1 to 1%, although it could be higher when considering 1-year mortality and/or considering societies with impaired health systems (2). In Ghana, the manner in which meats are handled by butchers in markets could easily expose the meats to Salmonella contamination (1, 3). This represents a health risk to Ghanaians since most of them consume locally produced animal meats on a regular basis as an important protein source.

In 2016, a total of 225 locally produced meat samples, namely, beef (*n* = 45), goat (*n* = 45), mutton (*n* = 45), guinea fowl (*n* = 45), and chicken (*n* = 45), were purchased from 5 retail shops in Tamale, the capital city of the northern region of Ghana. One hundred seven Salmonella enterica strains were isolated from these meat samples, according to the U.S. FDA bacteriological analytical manual, with slight modification (4). Briefly, meat samples (10 cm^2^) were swabbed and preenriched in buffered peptone water. Preenriched aliquots were further enriched in Rappaport-Vassiliadis and selenite cystine broths. The enriched aliquots were then streaked on xylose-lysine-deoxycholate and brilliant green agars. Presumptive Salmonella colonies were purified and confirmed by biochemical testing, Gram staining, and a *Salmonella* latex agglutination test (Oxoid Ltd., Basingstoke, UK). Overnight Luria-Bertani broth cultures of 16 selected isolates (beef [*n* = 3], goat [*n* = 3], mutton [*n* = 4], guinea fowl [*n* = 3], and chicken [*n* = 3]; Table 1) were subjected to DNA extraction using a QIAamp DNA minikit (Qiagen, Hilden, Germany). Library preparation was performed according to Illumina’s TruSeq Nano DNA sample preparation protocol, which was sequenced on the MiSeq platform (Illumina, CA, USA) with 300-bp paired-end read lengths (5). Raw reads were *de novo* assembled using the Shovill pipeline version 0.9.0 (https://github.com/tseemann/shovill) that uses SPAdes version 3.11.0, available in the GalaxyTrakr pipeline (https://www.galaxytrakr.org/ [6]). The “trim reads” option was selected, and the list of k-mer sizes to be used was set to “auto.” The draft genome assembly quality was evaluated using QUAST version 4.6.3 (7). Draft genomes were analyzed with the following Web-based tools from the Center for Genomic Epidemiology website (http://cge.cbs.dtu.dk/). PlasmidFinder version 2.0 (8) and ResFinder version 3.0 (9) were used to identify plasmid and antimicrobial resistance genes, respectively. MLST version 2.0 (10) and pMLST version 2.0 (8) were used to determine the multilocus sequence typing (MLST) profiles of the genome and plasmid, respectively. Raw reads of isolates with an unknown sequence type (ST) were submitted to EnteroBase (11) (https://enterobase.warwick.ac.uk/) for new ST assignment. *Salmonella* serovars were predicted from the draft genomes using SeqSero version 1.0 (http://www.denglab.info/SeqSero [12]).

**TABLE 1:** Whole-genome sequencing characterization of 16 nontyphoidal *Salmonella enterica* strains that were isolated from various meat samples in Ghana

Isolate no.	Laboratory identifier	Sample name	Sample type	MLST^*a*^	Predicted *S. enterica* subsp. *enterica* serovar(s)^*b*^	Plasmid replicon^*c*^	pMLST^*d*^		Genbank accession no.	No. of contigs (≥1,000 bp)^*e*^	Total length (bp) (≥1,000 bp)^*e*^	*N*_50_ (bp)^*e*^	GC content (%)^*e*^	Total no. of sequence reads^*f*^	Coverage (×)
							IncF	IncI1							
1	NAFTEC00104	AB11_S29	Beef	4605	Kaapstad				SIVZ00000000	19	4,565,905	714,420	52.17	1,165,102	70.1
2	NAFTEC00108	CB5_S22	Beef	2469	Lagos				SIWD00000000	18	4,763,790	728,760	52.25	1,205,222	72.6
3	NAFTEC00112	NB10_S20	Beef	2609	II 13,22:z:-				SIWH00000000	45	4,990,760	314,445	52.12	1,020,302	61.4
4	NAFTEC00105	AC3_S26^*g*^	Goat	5307	Ouakam				SIWA00000000	31	4,703,400	270,828	52.23	1,440,086	86.7
5	NAFTEC00109	CC5_S25	Goat	2469	Lagos				SIWE00000000	17	4,762,141	728,760	52.25	1,374,204	82.7
6	NAFTEC00113	NC6_S16	Goat	603	Infantis				SIWI00000000	40	4,617,319	208,256	52.3	782,636	47.1
7	NAFTEC00114	NLC13_S21^*g*^	Chicken	5308	Hato	IncI1		183^*h*^	SIWJ00000000	24	4,791,242	542,822	52.14	1,044,782	62.9
8	NAFTEC00117	SLC10_S19	Chicken	3899	Hato				SIWM00000000	17	4,695,615	708,046	52.18	1,290,122	77.7
9	NAFTEC00119	TLC7_S23^*g*^	Chicken	5308	Hato	IncI1		183^*h*^	SIWO00000000	20	4,792,827	583,712	52.14	940,136	56.6
10	NAFTEC00110	Cg4_S30^*g*^	Guinea fowl	5308	Hato	IncI1		183^*h*^	SIWF00000000	22	4,794,334	583,712	52.14	1,340,282	80.7
11	NAFTEC00116	Sg14_S27^*g*^	Guinea fowl	5308	Hato	IncI1		183^*h*^	SIWL00000000	22	4,794,271	583,712	52.13	1,358,892	81.8
12	NAFTEC00118	Tg14_S17^*g*^	Guinea fowl	5308	Hato	IncI1		183^*h*^	SIWN00000000	21	4,792,495	583,712	52.14	1,027,678	61.9
13	NAFTEC00107	AM10_S28^*g*^	Mutton	5307	Ouakam				SIWC00000000	31	4,704,550	270,828	52.23	1,276,258	76.8
14	NAFTEC00106	AM9_S6	Mutton	4605	Kaapstad				SIWB00000000	18	4,566,236	714,419	52.17	821,716	49.5
15	NAFTEC00111	CM7_S24	Mutton	101	Africana	IncFII(S)	[S1:A-:B-]		SIWG00000000	19	4,550,213	709,547	52.03	1,064,366	64.1
16	NAFTEC00115	NM14_S18	Mutton	4605	Kaapstad				SIWK00000000	17	4,565,451	714,419	52.17	1,107,944	66.7

a Using MLST version 2.0. MLST, multilocus sequence typing.

b Using SeqSero version 1.0.

c Using PlasmidFinder version 1.3 (minimum percentage identity, 95%; minimum length, 60%).

d Using pMLST version 2.0. pMLST, plasmid multilocus sequence typing.

e Using QUAST version 4.6.3.

f Sum of forward and reverse reads.

g Isolate with new ST being assigned by EnteroBase.

h Novel allele (i.e., allele with less than 100% identity is found); ST may indicate nearest ST.

The draft genomes ranged from 4,550,213 to 4,990,760 bp in size, with 52.2% average GC content (Table 1). The number of contigs for each isolate ranged from 17 to 45. Analysis by SeqSero revealed that the isolates belong to seven different serovars. It is noteworthy that all six isolates from poultry were Salmonella enterica subsp. enterica serovar Hato. Eight MLSTs were identified, including two that were newly assigned, ST5307 and ST5308. ResFinder identified only one antimicrobial resistance gene, *fosA7* (for fosfomycin), in a Salmonella enterica subsp. enterica serovar Africana strain of mutton origin. Only two plasmid replicon types belonging to IncI1 of *Salmonella* Hato of chicken and guinea fowl origin and IncFII(S) of *Salmonella* Africana of mutton origin were seen. The data provided will contribute to understanding the molecular diversity of Salmonella enterica strains found in retail meats in Tamale, the capital city of the northern region of Ghana. It will also be useful in comparative genomic analyses of Salmonella enterica from the meat production chain in Ghana, as well as those from humans when more of such sequence data are deposited into the public database in the future.

### Data availability.

The sequence data were deposited in GenBank under BioProject accession number PRJNA484344. GenBank accession numbers for individual isolates are listed in Table 1.
